# The MAPK and PI3K pathways mediate CNTF-induced neuronal survival and process outgrowth in hypothalamic organotypic cultures

**DOI:** 10.1007/s12079-015-0268-8

**Published:** 2015-02-20

**Authors:** Jason M. Askvig, John A. Watt

**Affiliations:** 1 Department of Biology, Concordia College, Moorhead, MN 56562 USA; 2 Department of Basic Sciences, University of North Dakota School of Medicine and Health Sciences, Room 1701 Stop 9037, 501 N Columbia Road, Grand Forks, ND 58203 USA

**Keywords:** PI3K, AKT, MAPK, STAT3, NFkB, CNTF, Axonal regeneration, Neuronal survival, Organotypic culture

## Abstract

While collateral sprouting has been shown to occur in a variety of neuronal populations, the factor or factors responsible for mediating the sprouting response remain largely un-defined. There is evidence indicating that ciliary neurotrophic factor (CNTF) may play an important role in promoting neuronal survival and process outgrowth in neuronal phenotypes tested to date. We previously demonstrated that the astrocytic Jak-STAT pathway is necessary to mediate CNTF-induced oxytocinergic (OT) neuronal survival; however, the mechanism (s) of CNTF-mediated process outgrowth remain unknown. Our working hypothesis is that CNTF mediates differential neuroprotective responses via different intracellular signal transduction pathways. In order to test this hypothesis, we utilized stationary hypothalamic organotypic cultures to assess the contribution of the MAPK-ERK and PI3-AKT pathways to OT neuron survival and process outgrowth. Our results demonstrate that the MAPK-ERK½ pathway mediates CNTF-induced neuronal survival. Moreover, we show that inhibition of the p38-, JNK-MAPK, and mTOR pathways prevents loss OT neurons following axotomy. We also provide quantitative evidence indicating that CNTF promotes process outgrowth of OT neurons via the PI3K-AKT pathway. Together, these data indicate that distinct intracellular signaling pathways mediate diverse neuroprotective processes in response to CNTF.

## Introduction

Collateral axonal sprouting has been shown to occur in a variety of neuronal populations within the central nervous system although the factors responsible for mediating axonal sprouting are still largely ill-defined. However, there is growing evidence indicating that ciliary neurotrophic factor (CNTF) plays an important role. For example, CNTF has been shown to promote axonal sprouting of hypothalamic magnocellular neurons in vitro (Askvig et al. 2013; Vutskits et al. 1998) and motor neuron sprouting in vivo (Gurney et al. 1992; Guthrie et al. 1997; Kwon and Gurney 1994; Oyesiku and Wigston 1996; Siegel et al. 2000; Simon et al. 2010; Ulenkate et al. 1994; Wright et al. 2007; Xu et al. 2009). Moreover, CNTF promotes the survival of magnocellular neurons in the paraventricular nucleus (PVN) and supraoptic nucleus (SON) of the magnocellular neurosecretory system in organotypic cultures (Askvig et al. 2013; House et al. 2009; Rusnak et al. 2002; Rusnak et al. 2003; Vutskits et al. 1998; Vutskits et al. 2003).

To induce a cellular response, CNTF first binds to a three-part receptor complex consisting of the ligand-specific binding subunit CNTF receptor alpha (CNTFRα), which is attached to the membrane via a glycosylphosphatidylinositol (GPI) linkage. The CNTF-CNTFRα complex then interacts with glycoprotein 130 (gp130) and leukemia inhibitory factor receptor beta (LIFRß) (Davis et al. 1993; Ip et al. 1993; Ip et al. 1992; Schuster et al. 2003) to form a functional transmembrane signaling complex. We have demonstrated previously that astrocytes within the SON are immunoreactive for CNTFRα, LIFRß, gp130 and CNTF protein (Askvig et al. 2012; Watt et al. 2006). However, magnocellular neurons are immunoreactive for CNTFRα and gp130 (Askvig et al. 2012; Watt et al. 2009) but not LIFRß indicating that the astrocytes but not magnocellular neurons of the SON are activated directly by CNTF (Davis et al. 1993). In support of this observation, we have shown that pressure injection of exogenous rat recombinant (rrCNTF) into the SON in vivo results in phosphorylation of STAT3 in astrocytes but not magnocellular neurons (Askvig et al. 2013). CNTF-induced activation of the Jak-STAT pathway mediates a significant increase in survival of axotomized magnocellular neurons in organotypic culture which was reduced by pharmacological inhibition of the Jak-STAT pathway (Askvig et al. 2013). In addition, we observed an extensive outgrowth of neuronal processes originating from oxytocinergic (OT) neurons in the SON and PVN in response to rrCNTF (Askvig et al. 2013). Together, these data indicate that CNTF-induced survival of OT magnocellular neurons is mediated through a paracrine interaction with astrocytes. Although the specific intracellular pathway (s) which mediate CNTF-induced process outgrowth remain unknown, others have demonstrated differential effects of the Jak-STAT, PI3-AKT and MAPK-ERK pathways in mediating neuronal survival versus process outgrowth (Alonzi et al. 2001; Dolcet et al. 2001; Ozog et al. 2008; Sango et al. 2008).

Our working hypothesis is that CNTF mediates neuronal survival and process outgrowth via different intracellular signal transduction pathways, respectively. In order to test this hypothesis, we utilized stationary hypothalamic organotypic cultures, as initially developed by Stoppini et al. (1991), to assess the contribution of the MAPK, NFkB and PI3-AKT pathways to magnocellular neuron survival and process outgrowth in the SON and PVN. Organotypic cultures exhibit several advantages over other in vitro culture systems primarily because of the preservation of the in vivo cytoarchitecture and the use of fully differentiated neurons (House et al. 1998; Vutskits et al. 1998). Furthermore, the ability to directly manipulate the culture media with growth factors and pharmacological agents and assess magnocellular neuron survival in hypothalamic organotypic cultures facilitates analysis of pathway-mediated cellular events more rapidly than can be achieved using in vivo injury model systems. However, as with all in vitro systems when interpreting experimental results consideration must be given to alteration of cellular activities specific to the tissue preparation and culture conditions used which may affect experimental outcomes. Nevertheless, stationary organotypic cultures provide a unique and powerful tool for exploring the factors and mechanisms through which specific central nervous systems respond to injury

## Materials and methods

### Animals

Pregnant Sprague Dawley rats (E15) were purchased from Harlan Laboratories (Minneapolis, MN). All rats were housed in the University of North Dakota Center for Biomedical Research Facility, an AAALAC accredited facility, under a 12 L:12D light cycle with ad lib access to lab chow and tap water throughout the investigations. Experimental protocols utilized in these studies followed the guidelines in the NIH guide for the care and use of laboratory animals and were approved by the UND Institutional Animal Care and Use Committee. All efforts were made to minimize the numbers of animals used in this study and their suffering.

### Stationary hypothalamic organotypic cultures

Organotypic cultures were prepared as previously described (Askvig et al. 2013). Briefly, 6-day-old Sprague Dawley rat pups were decapitated and their brains were removed and placed in chilled Geys Balanced Salt Solution (Gibco, Grand Island, NY) enriched with glucose (5 mg/ml; Sigma). The brains were then trimmed to remove exterior cortical material and 350 μm coronal sections obtained using a McIlwain Tissue Chopper (Stoelting). The sections containing the magnocellular neurosecretory system nuclei were placed in chilled Geys Balanced Salt Solution and then trimmed dorsal to the third ventricle and lateral to the SON under a dissecting microscope. Sections from each animal were then placed on a single Millicell-CM filter insert (pore size 0.4 μm, 30 mm diameter; Millipore, Bedford, MA) and each filter insert was then placed in a 35 × 10 mm Petri dish containing 1.1–1.2 ml of culture media for the experimental period.

### Media and incubations

The culture media was made fresh at the beginning of every experiment and consisted of Eagle’s Basal Medium with Earle’s salts (50 %; Gibco), heat inactivated horse serum (25 %; Gibco), Hank’s balanced salt solution (25 %; Gibco), glucose (0.5 %; Sigma), penicillin/streptomycin (25 units/ml; Gibco), and glutamine (1.0 mM; Gibco). The osmolality and pH of the culture media were measured from the stock media solution every 48 h using a Wescor vapor pressure osmometer (Wescor 5500; Logan, UT) and a mini pH meter (IQ Scientific Instruments, Loveland, CO), respectively. Our analysis demonstrated that the media osmolality was maintained at 310.5 ± 0.45 mOsm/l and the pH was at 8.2 ± 0.03 throughout the experimental period. Incubation of the cultures was stationary in 5 % CO_2_-enriched air at 35 °C for the entire experimental period.

Hypothalamic slices were cultured in the presence or absence of rrCNTF (#C3835, lot #080 M1730 or #091 M1403, Sigma) for 14 days. All groups had their culture media replaced every 48 h and always received fresh additions of rrCNTF. Inhibition experiments were performed by administering the inhibitor in the absence of rrCNTF for 1 h prior to treatment of the cultures followed by replacement with media containing the inhibitor plus rrCNTF for the duration of the experimental period. Additional control cultures received only the inhibitor for the entire experimental period. Multiple inhibitors, which had distinct mechanisms of action, were used to inhibit the pathways. The concentrations used for each inhibitor was determined from previous reports of others indicating optimal working concentrations of inhibitors used in primary cell cultures (Phulwani et al. 2008; Su et al. 2011; Wang et al. 2008), or when available, in organotypic cultures (Greenwood and Bushell 2010; Luo et al. 2007; Marwarha et al. 2010; Ohnishi et al. 2010; Rusnak and Gainer 2005; Zamin et al. 2006). Briefly, inhibition of various components of the MAPK pathway were inhibited with U0126, PD98059, PD184352, SP600125, and SB203580, the PI3K-AKT pathway was inhibited with LY294002 and wortmannin, mTOR signaling was inhibited utilizing rapamycin and torin-1, and the NFκB transcription factor was inhibited by bay 11-7082 and sc-514 (Table 1).

**Table 1:** Pharmacological inhibitors used in this study

Inhibitor (Concentration used)	Site of action	Source/Cat #
Bay 11-7082 (15, 30 μM)	IκBα	Calbiochem # 196870
LY294002 (15 μM)	PI3K	Calbiochem #440202
LY303511 (15 μM)	Negative control for LY294002.	Calbiochem # 440203
PD184352 (5 μM)	MEK½	Santa Cruz #sc-202759
PD98059 (5 μM)	MEK½ and MEK5	Calbiochem #513000
Rapamycin (10 μM)	mTORC1	Calbiochem #553210
SB203580 (75 μM)	p38 MAPK	Calbiochem # 559389
SC-514 (20 μM)	Iκκ-2(ß)	Calbiochem # 401479
SP600125 JNKII Inhibitor (50 μM)	c-Jun N-terminal kinase (JNK)	Calbiochem # 420119
Torin-1 (500 nM)	mTORC1 and mTORC2	Tocris (R&D Systems) #4247
U 0124 (1 μM)	Negative control for U 0126.	Calbiochem # 662006
U 0126 (1 μM)	MEK½ and MEK5	Calbiochem # 662005
Wortmannin (KY12420) (1 μM)	PI3K	Calbiochem # 681675

### Organotypic culture immunohistochemistry

Following experimental periods, the explants were prepared for immunohistochemistry with fixation in 4 % paraformaldehyde (Sigma) in 0.1 M phosphate buffer for 1.5 h. For immunohistochemical analysis, sections were washed with PBS-T in 3 × 10 min intervals before and after all incubations. For single-label peroxidase immunohistochemistry, endogenous peroxidase activity and non-specific staining were prevented by treatment with 0.3 % H_2_O_2_ (Sigma) followed by incubation in blocking buffer (10 % normal horse serum containing 0.3 % Triton X-100) for 1 h. The explants were then incubated for 36 h at 4 °C in a highly specific monoclonal mouse antibody against oxytocin (OT)-neurophysin (PS 38, 1:500; a gift from Dr. Harold Gainer). This antibody was first characterized by Ben-Barak et al. (1985) and has been shown to label specifically OT neurons and their processes within stationary organotypic cultures prepared from rat hypothalamus (House et al. 2009; House et al. 2006; House et al. 1998; Rusnak et al. 2002; Rusnak et al. 2003; Shahar et al. 2004; Vutskits et al. 1998; Vutskits et al. 2003). Next the cultures were incubated in horse anti-mouse biotinylated secondary antibody (1:500; Vector), followed by avidin-biotin complex (ABC; 10 μl/ml in PBS; Vector ABC *Elite* kit) for 1 h at room temperature. Bound antibodies were visualized using 0.05 % diaminobenzidine (DAB, Sigma) in PBS developed through the glucose-oxidase method (Itoh et al. 1979). The hypothalamic slices were then removed from their filters and placed directly on gelatin coated slides. All slides were then dehydrated in increasing concentrations of alcohol followed by xylene and coverslips mounted with Permount (Fisher, Pittsburgh, PA). All images were captured using an Olympus BX-51 light microscope with attached DP-71 color camera and dedicated software. Montage images were prepared for reproduction using the ‘photomerge’ option in Adobe Photoshop CS3.

### Magnocellular neuronal counts

The slides containing the immunoreactive explant culture slices were coded by a third party blind to the experimental conditions. In order to obtain the total number of neurons in the PVN and SON, immunoreactive cells were counted using a drawing tube attached to an Olympus BX51 microscope. The values used in statistical analysis represent the total number of immunoreactive neurons for each nuclei of one neonatal hypothalami (i.e., one filter insert) and it was the mean of two individual’s independent neuronal counts that were used as the group mean for statistical analysis as described below.

### Quantitative analysis of process density

In order to quantify the extent of OT-immunoreactive process outgrowth originating from the SON in organotypic cultures, we utilized a previously described stereological analysis technique first developed for quantification of neurite outgrowth from chick dorsal root ganglion (Bilsland et al. 1999) and modified for our culture system. Slides coded by a third party blind to the experimental conditions were viewed at 10× magnification using an Olympus BX51 microscope. In order to ensure consistency across groups, the SON was placed in the lower center frame of the picture (1360 × 1024 image size) and the frame digitally captured. MCID image analysis software (Cambridge, England) was used to quantify the area occupied by OT-immunoreactive processes using an automatic target detection and measurement feature that quantifies the area in pixels occupied by a target according to the defined target criteria (Bilsland et al. 1999). The target criteria are set combining the optical density and spatial characteristics of the target. Once the density was set, any pixel falling within this range in the image was automatically counted. A full image scan of the micrograph was performed which gave the proportional area of the entire micrograph that was occupied by OT-immunoreactivity (total proportional area). Next, the density was determined for the immunoreactive somata, which gave the somata proportional area. The somata proportional area was subtracted from the total proportional area to determine the proportional area that was occupied by OT-immunoreactive processes (process proportional area), which was the value that was utilized in the statistical analysis. As each organotypic culture differs in terms of intensity of immunoreactivity and background, the density was set for each image before quantification was undertaken. Since each SON varies in the amount of magnocellular neurons, we corrected for the number of neurons in the SON by standardizing the process proportional area to the total number of neurons in the SON. Thus, the values utilized for statistical analyses, as described below, represent the ratio of process proportional area to total number of neurons in the SON.

### Statistical analysis

Distribution normality of each group of data was tested using the Kolmogorov-Smirnov test (GraphPad InStat, version 3.06 for Windows; San Diego California) and all groups were normally distributed. Statistical differences between groups were compared using one-way ANOVA with Tukey’s post *hoc* test (GraphPad InStat) with *p < 0.05* considered statistically significant. Statistical values are reported in the appropriate figure legends. Results are expressed as the group means ± SD.

## Results

### The MAPK-ERK pathway mediates CNTF-induced OTneuronal survival in organotypic cultures

Three pharmacological inhibitors of the MAPK-ERK pathway were utilized; U0126, PD98059, and PD184352 (Table 1). All inhibit MAPKK (MEK), preventing activation of the immediate downstream target, ERK. U0126 and PD98059 are structurally related, and have been demonstrated to inhibit both MEK½ and MEK5 (Ballif and Blenis 2001; Su et al. 2011), while PD184352 specifically inhibits MEK½ (Bain et al. 2007). Organotypic cultures were also treated with U0124, which is a control molecule for the pharmacological inhibitor, U0126, and does not inhibit MEK activity even at concentrations of 100 μM (manufacturer’s technical sheet).

**Fig. 1:**
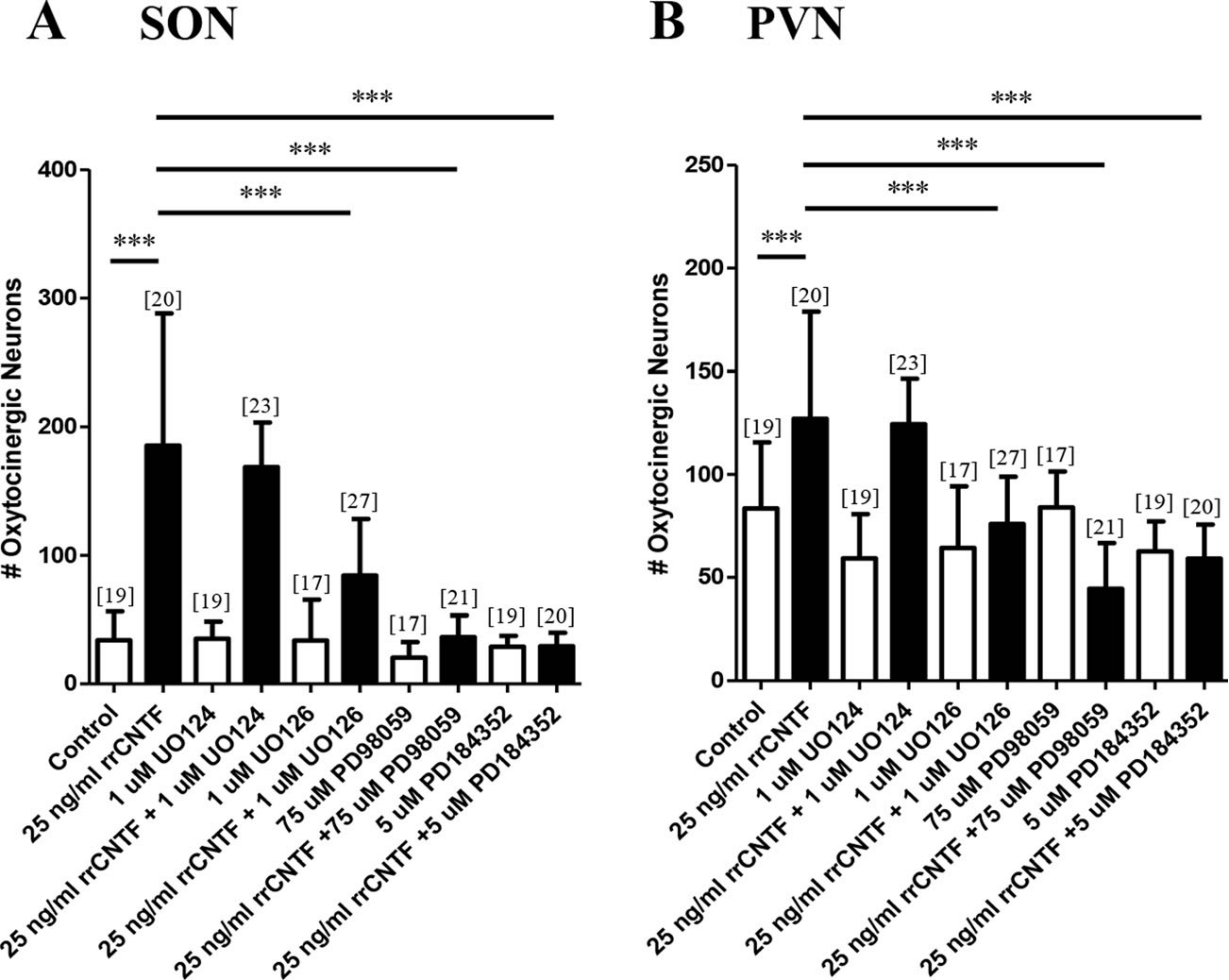
The MAPK-ERK½ pathway is necessary to mediate the CNTF-induced survival of OT neurons. Immunohistochemical neuronal cell counts demonstrated that exogenous rrCNTF promoted the survival of OT neurons (SON: *p < 0.0001;* PVN: *p < 0.0001*) compared to control, while inhibition of the MAPK-ERK½ pathway with U0126 (SON: *p < 0.0001*; PVN: *p < 0.0001*), PD98059 (SON: *p < 0.0001*; PVN: *p < 0.0001*), and PD184352 (SON: *p < 0.0001*; PVN: *p < 0.0001*) significantly reduced the number of surviving OT neurons in the SON (**a**) and PVN (**b**) compared to the 25 ng/ml rrCNTF group. Column bars and error bars represent the mean and SD of [n] groups. PVN, paraventricular nucleus; SON, supraoptic nucleus. ***p < 0.0001

Our results indicate that treatment of organotypic cultures with rrCNTF, (25 ng/ml, 14 days) (Askvig et al. 2013), resulted in a 443 % increase in the number of surviving OT neurons in the SON when compared with control values obtained from cultures maintained in rrCNTF-free media (Fig. 1a). Additionally, a significant 52 % increase in OT neuronal survival was also observed in the PVN (Fig. 1b).

When cultures were treated with 25 ng/ml rrCNTF in the presence of U0124, the control molecule, there was no significant difference in the number of surviving OT neurons in the SON or PVN (Fig. 1). In contrast, when the MEK½ and MEK5 pathways were inhibited with 1 μM U0126 in the presence of rrCNTF we observed a 55 and 40 % decrease in the number of surviving OT neurons in the SON and PVN, respectively (Fig. 1a and b). Likewise, CNTF treatment in the presence of PD98059 resulted in an 80 and 65 % decrease in the number of surviving OT neurons in the SON and PVN, respectively (Fig. 1b).

When cultures were treated with PD184352 in the presence of rrCNTF we observed an 84 and 53 % decrease in OT neurons in the SON and PVN, respectively (Fig. 1b). When organotypic cultures were treated with inhibitors alone there was no significant difference in the number of OT neurons in the SON or PVN (Fig. 1b) demonstrating that the inhibitors alone did not adversely affect OT neuron survival. Together, these data demonstrate that pharmacological inhibition of the MAPK pathway prevented CNTF-induced survival of OT neurons in the SON and PVN.

### JNK-MAPK and p38 do not mediate CNTF-induced neuronal survival

**Fig. 2:**
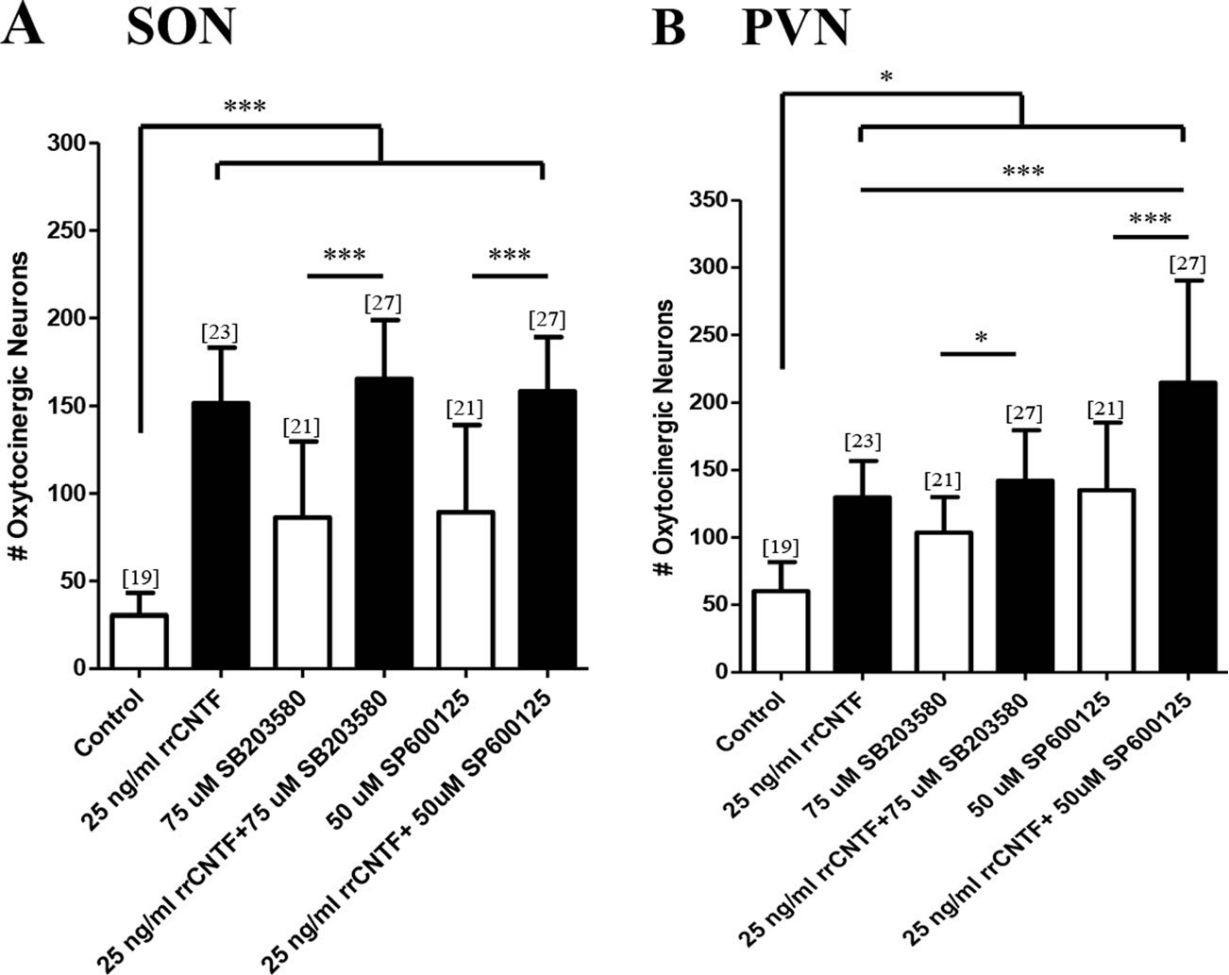
The p38- and JNK-MAPK pathways are not necessary to mediate the CNTF-induced survival of OT neurons. Immunohistochemical neuronal cell counts demonstrated that exogenous rrCNTF promoted the survival of OT neurons (SON: *p < 0.0001*; PVN: *p < 0.05*) compared to control, while inhibition of the p38- and JNK-MAPK pathways with SB203580 (SON: *p < 0.0001*; PVN: *p < 0.0001*) and SP600125 (SON: *p < 0.0001*; PVN: *p < 0.0001*), respectively, did not affect the number of surviving OT neurons in the SON (**a**) or PVN (**b**) compared to the 25 ng/ml rrCNTF group. However, when cultures were treated with the inhibitors alone, there was a statistically significant increase in the number of surviving OT neurons in the SON (A; SB203580: *p < 0.0001*; SP600125: *p < 0.0001*) and PVN (B; SB203580: *p < 0.05*; SP600125: *p < 0.0001*) compared to control. Column bars and error bars represent the mean and SD of [n] groups. *PVN* paraventricular nucleus, *SON* supraoptic nucleus. *p < 0.05, ***p < 0.0001

For the pharmacological inhibition of the p38-MAPK pathway, we utilized SB203580, which inhibits the p38α and ß isoforms, but not the γ and δ isoforms, and does not inhibit any of the JNK- or ERK-MAPK isoforms (English and Cobb 2002; Greenwood and Bushell 2010; Lee et al. 1994). Our analysis demonstrated that SB203580 in the presence of rrCNTF did not adversely affect neuronal survival in the SON or PVN (Fig. 2a and b). Conversely, treatment of cultures with inhibitor alone resulted in a 184 and 72 % increase in the number of OT neurons in the SON and PVN, respectively (Fig. 2a and b) suggesting that the p38-MAPK pathway mediates injury-induced cell death of OT neurons.

We next applied SP600125 (JNK Inhibitor II) which has equal potency towards all three of the JNK isoforms (JNK1-3) and cJun (Bennett et al. 2001; Ohnishi et al. 2010). Results indicate that SP600125 in the presence of rrCNTF did not adversely affect CNTF-induced neuron survival in the SON (Fig. 2a). However, in the PVN, we observed a 66 % increase in surviving OT neurons following treatment with SP600125 in the presence of rrCNTF. Treatment with SP600125 alone resulted in a 194 % increase in OT neuron survival the SON (Fig. 2a) and by 125 % in the PVN (Fig. 2b).

### The PI3K-AKT pathway mediates CNTF-induced process outgrowth

**Fig. 3:**
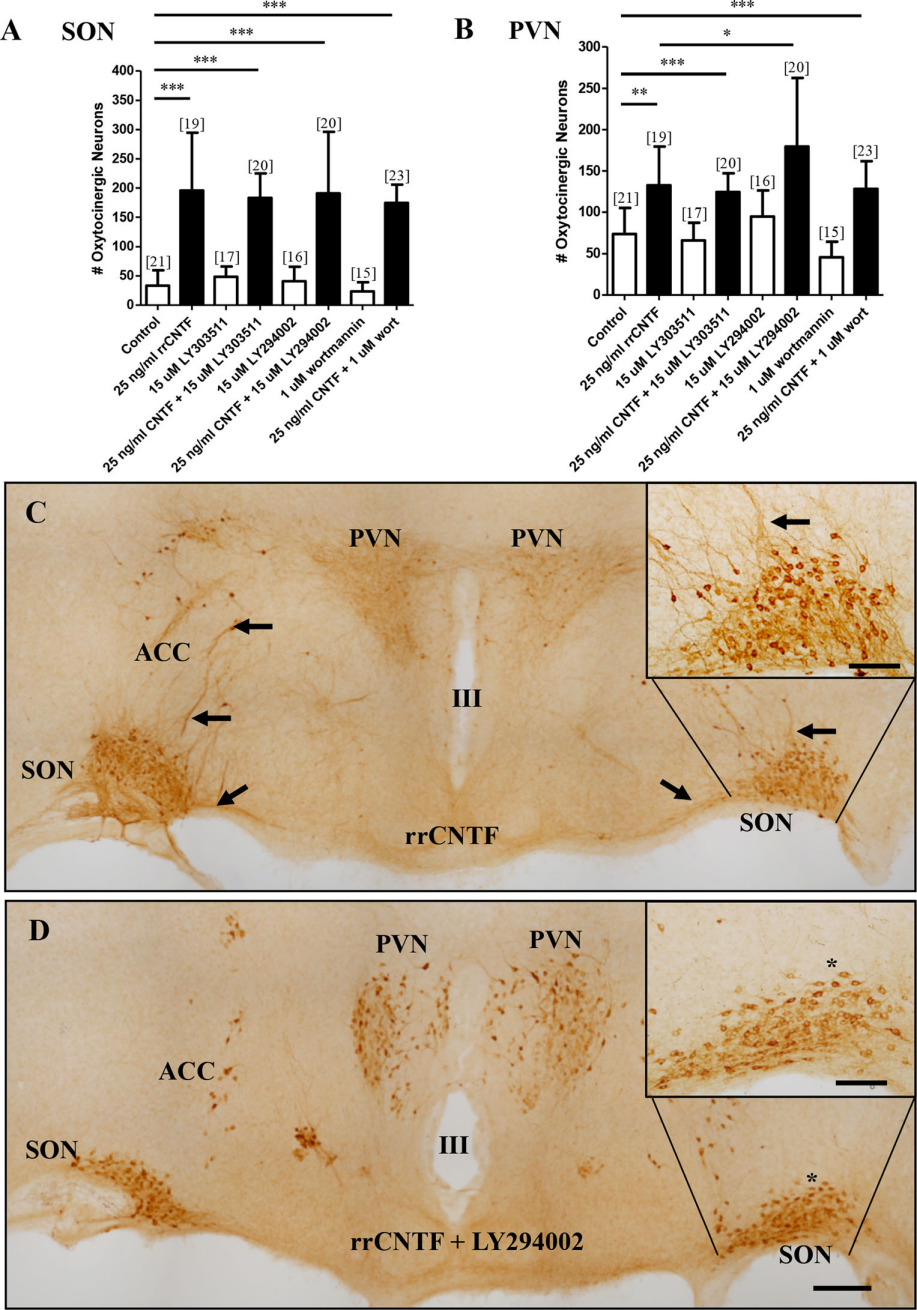
The PI3K-AKT pathway is not necessary to mediate the CNTF-induced survival of OT neurons. Immunohistochemical neuronal cell counts demonstrated that exogenous rrCNTF promoted the survival of OT neurons (SON: *p < 0.0001*; PVN: *p < 0.01*) compared to control, while inhibition of the PI3K-AKT pathway with LY294002 (SON: *p = 0.6014*; PVN: *p = 0.4931*) did not affect the number of surviving OT neurons in the SON (**a**) or PVN (**b**) compared to 25 ng/ml rrCNTF. In addition, the presence of wortmannin did not affect the number of surviving OT neurons in the SON (A; *p = 0.*8791); although there was a statistically significant increase in the number of surviving OT neurons in the PVN (B; *p = 0.0373*) compared to the 25 ng/ml rrCNTF group When visibly comparing the number of OT neurons between rrCNTF (**c**) and the rrCNTF plus LY294002 (**d**) groups, it is apparent that there are fewer neuronal processes present in the groups receiving PI3K inhibition compared to the rrCNTF group, even though they have the same number of surviving OT neurons (compare insets in C and D). Note that the representative images were obtained from approximately the same level of the magnocellular neurosecretory system, which is apparent when comparing the III ventricle between the images. Column bars and error bars represent the mean and SD of [n] groups. *ACC* accessory nuclei, *PVN* paraventricular nucleus, *SON* supraoptic nucleus. Scale bar C, D = 300 μm, inset = 100 μm. *p < 0.05, **p < 0.01, ***p < 0.0001

Two pharmacological inhibitors of PI3K were utilized; LY294002, which blocks the ATP binding site of PI3K (Zamin et al. 2006), and wortmannin (KY12420), which blocks the catalytic activity of PI3K (Luo et al. 2007). In addition, LY303511, which contains a single atom substitution in the morpholine ring and does not inhibit PI3K at concentrations up to 100 μM (manufacturer’s technical sheet) was used as a control for LY294002. We found that treatment with LY303511, LY294002 or wortmannin in the presence of rrCNTF did not result in a significant difference in the number of surviving OT neurons in the SON or PVN, although the number of surviving OT neurons were still significantly elevated from non-CNTF treated control (Fig. 3a and b). When organotypic cultures were treated with inhibitors alone there was no difference in the number of OT neurons in the SON or PVN. Together, these data demonstrate that pharmacological inhibition of the PI3K-AKT pathway does not affect CNTF-induced OT neuronal survival in hypothalamic organotypic explant cultures.

**Fig. 4:**
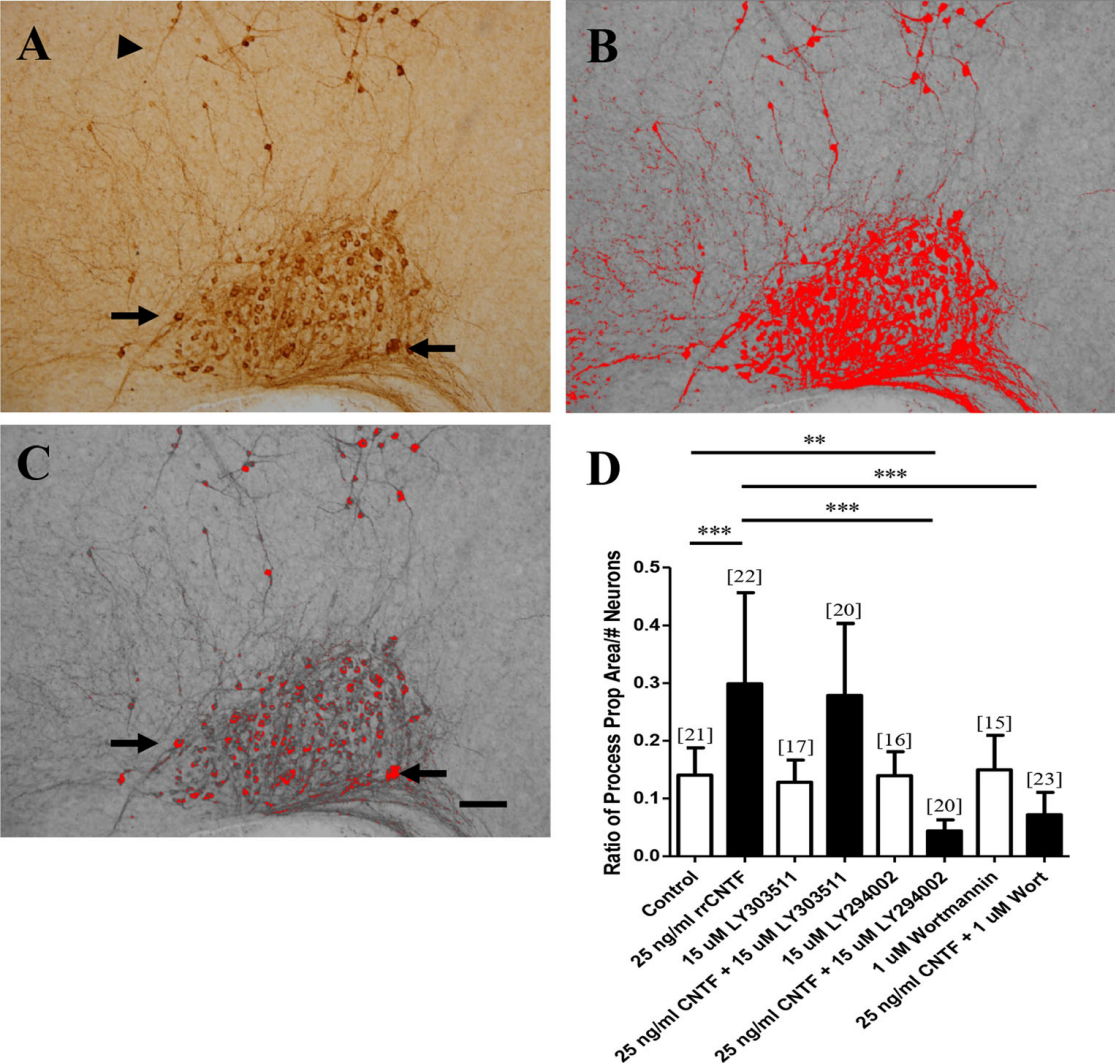
Inhibition of PI3K reduces the proportional area of OT-immunoreactive processes in the SON. **a** The image of the SON, captured such that the entire SON is centered in the bottom of the image. **b** A highlighted SON is shown following setting of the density. The density has been set to highlight the immunoreactive processes and somata, however, note that the analysis is a conservative estimate of the proportional area of neuronal processes because not all of the immunoreactive processes are highlighted (*arrowheads*, *A*, *B*). **c** A highlighted SON is shown following the setting of the density to highlight just the immunoreactive somata. Note that similar to their immunoreactive profiles, the nuclei are not highlighted by the density setting (*arrows*, *A*, *C*). **d** The ratio of proportional area of OT-immunoreactive processes to total number of neurons in the SON demonstrated that following administration of exogenous rrCNTF there was a significant increase of 112 % in the proportional area of OT-immunoreactive processes compared to control (*p < 0.0001*). The LY303511 molecule served as a control molecule for the LY294002 inhibitor and the proportional area of OT-immunoreactive processes in the LY303511 in conjunction with 25 ng/ml rrCNTF group did not differ from the 25 ng/ml rrCNTF group (*p = 0.6496*). However, when the PI3K inhibitors, LY294002 and wortmannin, were administered to the organotypic cultures in the presence of 25 ng/ml rrCNTF, there was a significant reduction in the proportional area of OT-immunoreactive processes in the SON compared to the 25 ng/ml rrCNTF group (*p < 0.0001*). Column bars and error bars represent the mean and SD of [n] groups. Scale bar = 100 μm. **p < 0.01, ***p < 0.0001

When visibly comparing the number of OT neurons between cultures treated with rrCNTF (Fig. 3c and inset), and rrCNTF plus LY294002 (Fig. 3d and inset), it is apparent that although there are similar numbers of OT neurons present there are fewer neuronal processes present in the groups receiving PI3K inhibition. Therefore, we utilized quantitative optical densitometric stereological analysis to determine the proportional area of OT-immunoreactive processes in the SON. Figure 4 illustrates the procedure. The value utilized in the statistical analysis was the ratio of the process proportional area to the total number of neurons in the SON. Our results demonstrate that following administration of exogenous rrCNTF there was a 112 % increase in the proportional area of OT-immunoreactive processes compared to control (Fig. 4d). LY303511 plus rrCNTF group did not differ from the rrCNTF group (Fig. 4d). However, when the PI3K inhibitors, LY294002 and wortmannin, were administered to the organotypic cultures in the presence of rrCNTF, there was a significant reduction in the proportional area of OT-immunoreactive processes in the SON (Fig. 4d). Moreover, the LY294002 plus rrCNTF group was significantly reduced from control values (Fig. 4d). These observations demonstrate that while PI3K signaling does not influence CNTF-mediated neuronal survival, it does mediate CNTF-induced process outgrowth of OT magnocellular neurons in the SON.

### mTORC1 and C2 do not mediate CNTF-induced neuronal survival or process outgrowth

**Fig. 5:**
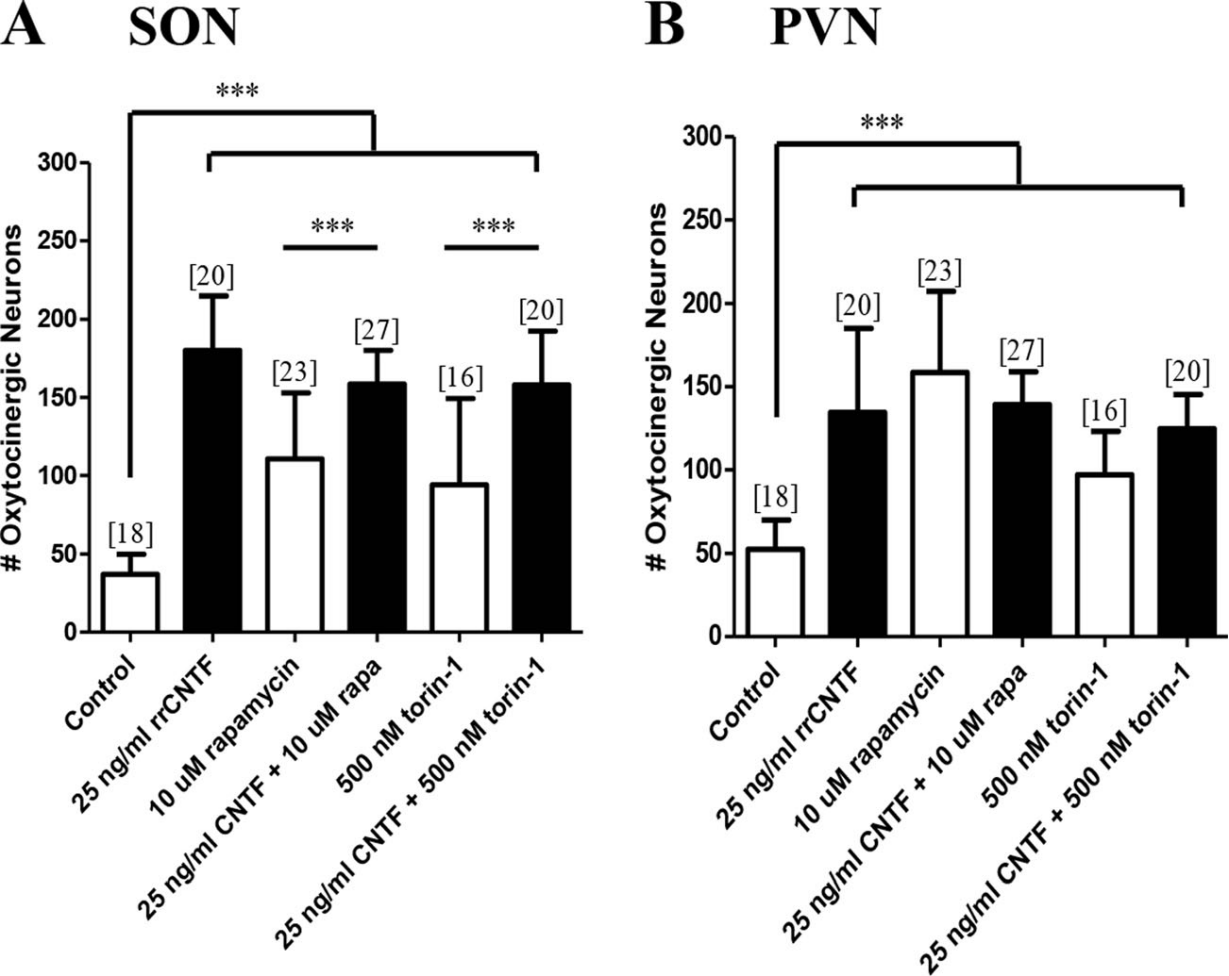
mTOR is not necessary to mediate CNTF-induced survival of OT neurons. Immunohistochemical neuronal cell counts demonstrated that exogenous rrCNTF promoted the survival of OT neurons (SON: *p < 0.0001*; PVN: *p < 0.0001*) compared to control, while inhibition of mTOR with rapamycin (SON: *p = 0.1113*; PVN: *p = 0.6633*) and torin-1 (SON: *p = 0.1487*; PVN: *p = 0.4250*) did not affect the number of surviving OT neurons in the SON (**a**) or PVN (**b**) compared to 25 ng/ml rrCNTF. However, when cultures were treated with the inhibitors alone, there was a significant increase in the number of surviving OT neurons in the SON (A; rapamycin: *p < 0.0001*; torin-1: *p < 0.0001*) and PVN (B; rapamycin: *p < 0.0001*; torin-1: *p < 0.0001*) compared to control. Column bars and error bars represent the mean and SD of [n] groups. *PVN* paraventricular nucleus, *SON* supraoptic nucleus. ***p < 0.0001

Rapamycin is a selective and potent inhibitor of mTOR complex 1 (mTORC1) (Marwarha et al. 2010), we also utilized torin-1, which is a selective and potent inhibitor for both mTORC1 and mTORC2 (Thoreen et al. 2012; Thoreen et al. 2009; Thoreen and Sabatini 2009). Moreover, torin-1 has been demonstrated to provide more complete inhibition of mTORC1 when compared to rapamycin (Thoreen and Sabatini 2009). Our analysis demonstrated that 10 μM rapamycin or 500 nM torin-1 in the presence of rrCNTF did not result in a statistically significant difference in the number of surviving OT neurons in the SON or PVN (Fig. 5a and b).

When organotypic cultures were treated with rapamycin or torin-1, in the absence of rrCNTF, there was a statistically significant increase in the number of OT neurons in the SON and PVN. These results suggest that mTOR signaling may be involved in the injury-induced cell death process in hypothalamic organotypic cultures. However, in contrast to inhibition of PI3K, quantitative optical densitometric analysis demonstrated that pharmacological inhibition of mTOR with rapamycin or torin-1 did not result in a statistically significant difference in the proportional area of OT-immunoreactive processes in the SON (data not shown).

### NF-κB does not mediate CNTF-induced neuronal survival or process outgrowth

**Fig. 6:**
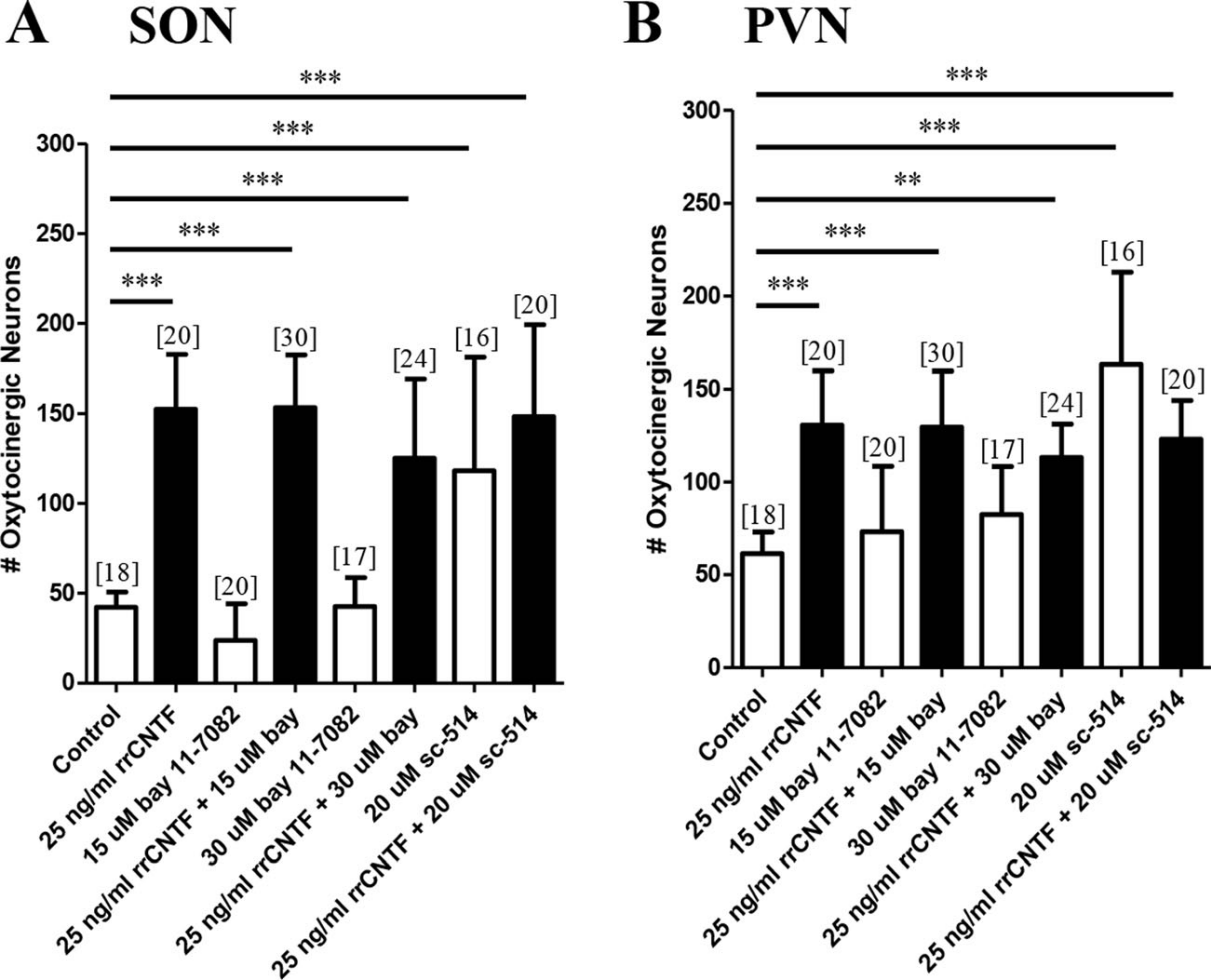
NF-κB is not necessary to mediate the CNTF-induced survival of OT neurons. Immunohistochemical neuronal cell counts demonstrated that exogenous rrCNTF promoted the survival of OT neurons (SON: *p < 0.0001*; PVN: *p < 0.0001*) compared to control, while inhibition of NF-κB with bay 11-7082 (15 μM: SON: *p = 0.9228*; PVN: *p = 0.9003;* 30 μM: SON: *p = 0.0828*; PVN: *p = 0.0667*) and sc-514 (SON: *p = 0.7561*; PVN: *p = 0.3522*) did not affect the number of surviving OT neurons in the SON (**a**) or PVN (**b**). However, when cultures were treated with sc-514 alone, there was a statistically significant increase in the number of surviving OT neurons in the SON (A; *p < 0.0001*) and PVN (B; *p < 0.0001*), compared to control. Column bars and error bars represent the mean and SD of [n] groups. *PVN* paraventricular nucleus, *SON* supraoptic nucleus. **p < 0.01, ***p < 0.0001

We utilized two pharmacological agents to inhibit NF-κB. BAY 11-7082, acts by inhibiting IκBα phosphorylation (Phulwani et al. 2008) while SC-514 targets the IKK complex which consists of three subcomponents, IKKα , IKKβ , and IKKγ. SC-514 highly specific for the IKKβ component and shows little effect on the other isoforms in vitro . Our analysis demonstrated that neither bay 11-7082 nor sc-514 in the presence of 25 ng/ml rrCNTF resulted in a significant difference in the number of surviving OT neurons in the SON or PVN (Fig. 6a and b). Moreover, quantitative analysis demonstrated that pharmacological inhibition of NF-κB with bay 11-7082 did not result in a significant difference in the proportional area of OT-immunoreactive processes in the SON compared to the 25 ng/ml rrCNTF group (data not shown). When organotypic cultures were treated with bay 11-7082 in the absence of rrCNTF there was no difference in the number of OT neurons in the SON. In contrast, when organotypic cultures were treated with sc-514 in the absence of rrCNTF a 181 and 166 % increase in the number of OT neurons in the SON and PVN, respectively was observed indicating that activation of the NF-κB complex via a specific IKKβ component may promote neuronal loss.

## Discussion

Our results indicate that MAPK, PI3-Akt and Jak-Stat pathways are activated in response to exogenous CNTF and these pathways mediate differentially neuronal survival and axonal regeneration by magnocellular neurons. While the Jak-STAT pathway is considered the canonical CNTF signal transduction pathway, CNTF has also been demonstrated to activate other intracellular signaling pathways including, MAPK, PI3K, and NFkB (Bonni et al. 1993; Cagnon and Braissant 2009; Dolcet et al. 2001; Gallagher et al. 2007; Kassen et al. 2009; Loy et al. 2011; Lutticken et al. 1994; Muller et al. 2009; Park et al. 2004; Peterson et al. 2000; Rhee et al. 2004; Sango et al. 2008; Symes et al. 1994; Trimarchi et al. 2009). For example, CNTF activates the MAPK pathway via SH2 domain-containing proteins, including SHP2 and Shc, which are bound to the LIFR and gp130 components of the CNTF receptor complex (Giordano et al. 1997; Stahl et al. 1995). Consistent with our findings others have demonstrated that the MAPK-ERK½ pathway promotes neuronal survival following injury (Chicoine and Bahr 2007; Jover-Mengual et al. 2007; Nagata 1999), primarily through the inhibition of the pro-apoptotic molecule BAD and production of the anti-apoptotic molecules, Bcl-2 and Bcl-x_L_ (Nagata 1999). Our studies extend these observations by demonstrating that the MAPK-ERK½ pathway mediates CNTF-induced neuronal survival in the magnocellular neurosecretory system in vitro. Functional activation of the MAPK-ERK½ pathway may occur via two potential mechanisms. First, unlike Jak molecules, which are tyrosine kinases, members of the MAPK pathway are serine/threonine kinases. Thus, MAPK-dependent activation of STAT3 occurs at Ser^727^ as opposed to the more commonly Jak-dependently phosphorylated Tyr^705^ (Decker and Kovarik 2000). However, following pressure injection of exogenous rrCNTF in to the SON, we observed STAT3 activation specifically at the Tyr^705^ residue (Askvig et al. 2013) indicating that MAPK-ERK½ pathway is not upstream of STAT3 activation in the astrocytes. Alternatively, activation of the Jak-STAT pathway may lead to activation of the MAPK-ERK½ pathway (Frank et al. 1995; Ihle and Kerr 1995; Winston and Hunter 1996). This mechanism appears to be dependent on Jak-mediated Ras or Raf activation of ERK½. While Jak-mediated activation of STAT molecules has been reported to be Ras-independent, Jak-mediated activation of the MAPK-ERK½ pathway has been demonstrated to be Ras-dependent (Winston and Hunter 1995). Others have demonstrated that Raf physically associates with Jak2, and Raf is tyrosine phosphorylated when co-expressed with Jak2 (Xia et al. 1996) raising the possibility of CNTF activation of both pathways in parallel following exogenous CNTF.

### The role of the p38-, JNK-MAPK, and mTOR pathways in injury-induced neuronal death

The p38- and JNK-MAPK pathways are structurally similar, but functionally distinct, from the classic MAPKs (ERKs). These pathways are preferentially activated by environmental stresses such as UV radiation, heat and osmotic shock, and by pro-inflammatory cytokines, such as tumor necrosis factor (TNF) and interleukin-1 (IL-1) (Tibbles and Woodgett 1999). Others have demonstrated that CNTF, which is classified as a pro-inflammatory cytokine, is capable of activating the p38- (Loy et al. 2011) and the JNK-MAPK pathways (Cagnon and Braissant 2009). Pharmacological inhibition of the p38- and JNK-MAPK pathways did not affect CNTF-mediated neuronal survival in hypothalamic organotypic cultures. However, unexpectedly, when the organotypic cultures were cultured only in the presence of the inhibitors, there was an increase in the survival of OT neurons. Similarly, when the organotypic cultures were cultured only in the presence of the mTOR inhibitors we observed a similar increase in the survival of OT neurons. These data suggest that the p38, JNK, and mTOR signaling components may mediate the post-axotomy responses that lead to neuronal death in the organotypic cultures.

Magnocellular vasopressinergic neurons in organotypic cultures of the PVN undergo neuronal death via apoptosis (Vutskits et al. 1998). Preparation of the organotypic cultures used in these studies resulted in a loss of greater than 90 % of OT neurons (Askvig et al. 2013). Furthermore, the anti-apoptotic agents, Bcl-x_L_, and Z-VAD-fmk protect both OT and VP magnocellular neurons in the SON of organotypic cultures, (House et al. 2006). These data suggest that the loss of magnocellular neurons observed in our cultures is likely due to apoptosis. A well documented function of the p38-MAPK and JNK-MAPK pathways is their role in the onset of apoptosis (Cuenda and Rousseau 2007; Dhanasekaran and Reddy 2008). Moreover, because of the overlap of the MAPKKs, specifically MKK4 (Cargnello and Roux 2011), in the p38-MAPK and JNK-MAPK pathways, these pathways could be activated by the same stimulus. Thus, our data suggest that the enhanced survival of the OT neurons that we observed in the presence of the p38 and JNK inhibitors alone results from pharmacological inhibition of the apoptotic cascade.

We show that inhibition of mTOR also protects injured OT neurons in the absence of exogenous rrCNTF. While mTOR has been primarily linked to the PI3K-AKT pathway, numerous reports have demonstrated mTOR signaling in the MAPK pathways (Chen et al. 2008; Chen et al. 2011; Karassek et al. 2010; Kato et al. 2012; Miller et al. 2007; Xu et al. 2011). The primary functions of PI3K-AKT-mTOR signaling have been attributed to promoting process outgrowth, cell survival, proliferation and growth (Chen et al. 2008; Chen et al. 2011; Karassek et al. 2010; Kato et al. 2012; Miller et al. 2007; Xu et al. 2011; Zhou and Huang 2010). Paradoxically, mTORC1 signaling has also been shown to mediate apoptosis through inhibition of AKT and selective activation of the JNK-MAPK pathway (Kato et al. 2012). These reports suggest that JNK is capable of mediating an apoptotic response via mTOR, leading to our hypothesis that following the axotomy during the organotypic culture preparation the p38- and JNK-MAPK pathways were co-activated to induce an apoptotic cascade in the OT neurons through the downstream signaling of mTOR.

Within the magnocellular neurosecretory system, understanding the specific mechanisms that regulate injury-induced neuronal apoptosis has not been well characterized. These novel data provide valuable insight into the mechanisms of axotomy-induced cell death that may lead to therapies that promote neuronal survival. Until future in vivo analyses can be performed, the full therapeutic potential remains unclear.

### The role of the PI3K-AKT pathway in CNTF-induced process outgrowth

In addition to activating the Jak-STAT and MAPK pathways, reports have also demonstrated that CNTF activates the PI3K-AKT pathway (Dolcet et al. 2001; Gold et al. 1994; Oh et al. 1998) through a Jak-dependent mechanism (Dolcet et al. 2001). Pharmacological inhibition of PI3K did not affect CNTF-induced neuronal survival, indicating that CNTF does not utilize PI3K signaling to promote OT neuronal survival in the magnocellular neurosecretory system. Interestingly, there was a remarkable decrease in process outgrowth in the SON following pharmacological inhibition of PI3K compared to the CNTF-treated cultures. Although the tissue thickness and complexity of the processes prevented stereological analysis of aspects of neurite outgrowth such as elongation, branching, and neurite caliber, we utilized quantitative optical densitometric stereological analysis to determine the proportional area of OT-immunoreactive processes which was corrected for the total number of neurons in the SON. This analysis resulted in a quantitative, yet conservative, analysis of process-immunoreactivity which confirmed our visual observation that there were less OT-immunoreactive processes present in the SON following PI3K inhibition. Thus, these data demonstrate that PI3K signaling is involved in regulating CNTF-mediated process outgrowth, but not involved in mediating neuronal survival.

It is not surprising that diverse intracellular signaling pathways mediate distinct neuroprotective processes in response to CNTF. Numerous reports have demonstrated divergent pathway mediation of CNTF-induced neuronal survival and process outgrowth (Dolcet et al. 2001; Ozog et al. 2008; Park et al. 2004; Sango et al. 2008). Similarities between those reports and our own observations indicate that the PI3K-AKT pathway plays a predominant role in mediating CNTF-induced process outgrowth in a variety of neuronal phenotypes. The PI3K-AKT pathway has been well-documented to promote neuronal survival, however, more recently the PI3K-AKT pathway has been revealed to be a key regulator in several aspects of process outgrowth, including elongation, branching, and neurite caliber (Read and Gorman 2009). There are many signaling factors downstream of PI3K that have been demonstrated to influence process outgrowth, including; mTORC1 (Asnaghi et al. 2004; Nave et al. 1999) and NF-κB (Kane et al. 1999; Romashkova and Makarov 1999). Not surprisingly, the transcriptional products of the PI3K-AKT pathway that directly mediate process outgrowth are cytoskeletal elements, such as microtubules (Kobayashi et al. 1997).

mTOR functions as two distinct signaling complexes, mTORC1 and mTORC2. These two complexes consist of unique mTOR-interacting proteins which determine their substrate specificity (Zhou and Huang 2010). Interestingly, the roles of the mTOR complexes in the PI3K-AKT pathway are quite distinct. It is believed that mTORC2 functions upstream of AKT to assist in regulating maximal activity of AKT (Hresko and Mueckler 2005; Sarbassov et al. 2005), while mTORC1 is a downstream regulator of AKT activity (Zhou and Huang 2010). It was demonstrated that PI3K-AKT-mTOR signaling has been shown to promote growth and branching of hippocampal neurons (Jaworski et al. 2005). Our analysis demonstrated that pharmacological inhibition of mTORC1 and mTORC2 did not affect OT neuronal survival or the proportional area of OT-immunoreactive processes, suggesting that mTOR is not involved in CNTF-mediated PI3K-AKT dependent process outgrowth of OT neurons.

NF-κB has also been implicated in mediating process outgrowth downstream of the PI3K-AKT pathway (Armstrong et al. 2008; Gutierrez et al. 2005; O’Neill and Kaltschmidt 1997; Sole et al. 2004) therefore we sought to determine its potential role in mediating neuronal survival and process outgrowth in our system. Although our results show that inhibition of BAY-11-7082 and SC-514 had no affect on process outgrowth we did observe that inhibition of the IKKβ complex specifically and in the absence of CNTF resulted in a significant increase in cell survival in both the SON and PVN. Increased NF-κB activation have been observed in neurons and astrocytes following CNS injury and evidence suggests a dual role for NF-κB in neuronal survival versus neuronal degeneration depending on the type of stimulus or cell phenotype activated (Mattson). Indeed, consistent with our observations, selective inactivation of NF-κB in astrocytes leads to increased survival of retinal ganglion cells following ischemia and promotes neuronal survival and axonal sprouting of central motor neurons following spinal cord injury (Brambilla2005.2009).

## Conclusions

We and others have demonstrated previously that exogenous CNTF will induce magnocellular neuron sprouting in vitro (Askvig et al. 2013; Vutskits et al. 1998). However, the cellular signaling pathways mediating this event were not identified. In this study we have extended these observations to show that the PI3K pathway mediates CNTF-induced OT-process outgrowth in the SON. We also demonstrate here that the MAPK-ERK½ pathway promotes CNTF-induced neuronal survival. Furthermore, we show that the p38-, JNK-MAPK, mTOR and NF-κB pathways promote magnocellular neuronal survival in the absence of CNTF following axotomy. Taken together, these data indicate that distinct intracellular signaling pathways mediate diverse neuroprotective processes in response to CNTF. The results of these studies will provide the basis for future studies that will continue to elucidate the mechanisms underlying CNTF-mediated neuroprotection and axonal regeneration in the CNS. Our long-term goal is development of therapeutic strategies that will allow selective regulation of axonal sprouting and promote neuronal survival following injuries or disorders that result in neurodegeneration.

## Abbreviations

CNTF: Ciliary neurotrophic factor
CNTFRα: Ciliary neurotrophic factor receptor alpha
gp130: Glycoprotein 130
LIFRß: Leukocyte inhibitory factor receptor beta
OT: Oxytocinergic
PVN: Paraventricular nucleus
SON: Supraoptic nucleus
VP: Vasopressinergic
